# Metformin as an adjuvant treatment for cancer: a systematic review and meta-analysis

**DOI:** 10.1093/annonc/mdw410

**Published:** 2016-09-28

**Authors:** C. Coyle, F. H. Cafferty, C. Vale, R. E. Langley

**Affiliations:** MRC Clinical Trials Unit at University College London, London, UK

**Keywords:** metformin, repurposing, adjuvant, prostate cancer, colorectal cancer, breast cancer

## Abstract

**Background:**

Metformin use has been associated with a reduced risk of developing cancer and an improvement in overall cancer survival rates in meta-analyses, but, to date, evidence to support the use of metformin as an adjuvant therapy in individual cancer types has not been presented.

**Patients and methods:**

We systematically searched research databases, conference abstracts and trial registries for any studies reporting cancer outcomes for individual tumour types in metformin users compared with non-users, and extracted data on patients with early-stage cancer. Studies were assessed for design and quality, and a meta-analysis was conducted to quantify the adjuvant effect of metformin on recurrence-free survival (RFS), overall survival (OS) and cancer-specific survival (CSS), to inform future trial design.

**Results:**

Of 7670 articles screened, 27 eligible studies were identified comprising 24 178 participants, all enrolled in observational studies. In those with early-stage colorectal cancer, metformin use was associated with a significant benefit in all outcomes [RFS hazard ratio (HR) 0.63, 95% confidence interval (CI) 0.47–0.85; OS HR 0.69, CI 0.58–0.83; CSS HR 0.58, CI 0.39–0.86]. For men with early-stage prostate cancer, metformin was associated with significant, or borderline significant, benefits in all outcomes (RFS HR 0.83, CI 0.69–1.00; OS HR 0.82, CI 0.73–0.93; CSS HR 0.58, CI 0.37–0.93); however, there was significant heterogeneity between studies. The data suggest that prostate cancer patients treated with radical radiotherapy may benefit more from metformin (RFS HR 0.45, CI 0.29–0.70). In breast and urothelial cancer, no significant benefits were identified. Sufficient data were not available to conduct analyses on the impact of metformin dose and duration.

**Conclusions:**

Our findings suggest that metformin could be a useful adjuvant agent, with the greatest benefits seen in colorectal and prostate cancer, particularly in those receiving radical radiotherapy, and randomised, controlled trials which investigate dose and duration, alongside efficacy, are advocated.

## Abstract (precis)

This systematic review and meta-analysis is the first to evaluate the evidence for an association between metformin use and cancer outcomes in patients undergoing treatment with curative intent for individual cancer types. Our findings suggest that adjuvant metformin could have beneficial effects, particularly on cancer outcomes in colorectal and prostate cancer. Randomised trials are warranted.

## introduction

Although cancer survival rates in the UK have doubled in the last 40 years, half of those diagnosed with cancer still die from their disease within 10 years [1, 2]. Adjuvant treatment after potentially curative cancer therapy improves survival rates, but relapse rates remain high in some tumour types, and for others, there are no proven adjuvant treatments. In the quest to improve cancer outcomes, a number of established medications with known anti-cancer properties have been considered as adjuvant anti-cancer therapies. Examples include aspirin [3], vitamin D [4], bisphosphonates [5], statins [6] and metformin.

Metformin exhibits a number of attributes that make it appealing for repurposing as an anti-cancer therapy. It has been in use for over half a century and is the most widely prescribed anti-diabetic medication in the world [7]. Consequently, it has been administered alongside most cancer treatments without the emergence of any important interactions. Additionally, data on the toxicity profile of metformin in those without type II diabetes mellitus (DM) are already available from clinical trials investigating its role as a treatment for polycystic ovarian syndrome [8]. Metformin is also generically available worldwide at low cost.

Metformin has been shown to have anti-cancer activity both *in vivo* and *in vitro* [9], with the underlying mechanism subject to ongoing investigation. It has been proposed that the anti-cancer properties of metformin result from both direct effects on cancer cells, particularly through inhibition of the AMPK/mTOR pathway [10], and indirect effects on the host, by virtue of its blood glucose-lowering properties and anti-inflammatory effects [11, 12]. Both mechanisms are anticipated to be important, although their relative contribution may differ according to cancer stage. *In vivo* evidence has emerged from window studies showing an anti-proliferative effect in breast cancer [13, 14] and a reduction in precancerous changes in the colorectum [15]. Meta-analyses have examined the role of metformin in the primary prevention of cancer, where it was found to significantly reduce overall cancer incidence; however, findings were inconsistent when individual tumour types were considered [16–20]. Meta-analyses have also investigated the effect of metformin use across all stages of disease and have found that it reduces overall cancer mortality rates, but, again findings are conflicting for individual tumour types [21–28], suggesting analyses are best conduced for individual tumour types separately. Most recently, a randomised phase III trial of non-DM patients showed that low-dose metformin was effective in the chemoprevention of metachronous colorectal adenomas or polyps when compared with placebo [29].

Benefits in the primary prevention, or advanced setting, do not necessarily translate to utility in the adjuvant setting as the mechanism of action may be different. Our objective was to conduct a systematic review and meta-analysis of randomised and non-randomised studies to investigate the effect of metformin use compared with non-use on recurrence-free survival (RFS), overall survival (OS) and cancer-specific survival (CSS) in adults who have potentially curable solid tumours. There have been a number of calls for systematic reviews and meta-analyses to be conducted as part of the scientific justification, and to inform the design, of new clinical trials [30, 31]. This is particularly relevant in the field of drug repurposing. The aim of this analysis was to advise further clinical investigation of metformin in the adjuvant setting.

## methods

All methods for this systematic review and meta-analysis are outlined in a prospectively registered protocol available online [32] (PROSPERO identifier CRD42015020519), and reporting follows PRISMA (Preferred Reporting Items for Systematic Reviews and Meta-Analyses) guidelines.

### eligibility criteria

Eligible studies include randomised, controlled trials and non-randomised studies (observational, cohort and case–control) that have investigated the use of metformin, with a comparator of no metformin, in participants over 16 years old with potentially curable solid tumours (defined as those either undergoing radical therapy with curative intent or those with an early-stage cancer where cure is normally the objective of standard treatment). Studies must have reported data on at least one of RFS, CSS or OS for individual tumour types.

### search strategy

Electronic searches of databases (Medline, EMBASE, Cochrane Central Register of Controlled Trials), clinical trial registries (clinicaltrials.gov, ISRCTN and EU Clinical Trials Register) and conference proceedings (American Society of Clinical Oncology, and European Society of Medical Oncology) were conducted. All sources were searched from inception until 31 May 2015 (conference abstracts 2005–2015). Bibliographies of the reports of all identified studies and review articles were hand-searched for further potentially eligible studies. Further details of the search strategy are available in supplementary data S1, available at *Annals of Oncology* online.

### study selection

All retrieved studies were assessed for eligibility and, when sufficient information was not available from the title and/or abstract, the full-text publication or (for conference abstracts) the associated poster or presentation was acquired and where this was not available, we contacted the study author. For studies with multiple publications, or where there was overlap in the patients studied, the most recent publication was chosen. Any queries were checked by a second reviewer and resolved by consensus. No study was excluded for weakness of study design or quality. For the purpose of this analysis, studies presenting data separately by tumour type were treated as separate studies. Articles were grouped by cancer type according to the site of origin and histology.

### data items and collection

Data on patient characteristics, interventions and outcomes were extracted for all studies into a predesigned table. These were cross-checked by a second independent reviewer and any disagreements were resolved by consensus. A list of data extracted is available in supplementary data S2, available at *Annals of Oncology* online. Studies were evaluated to determine whether they accounted for potential confounding factors [body mass index (BMI), age, gender, cancer-specific prognostic factors and the use of other anti-DM medications], either by demonstrating that there was no significant difference in their distribution between treatment groups or by inclusion in multivariable analyses. In order to minimise the potential for confounding by DM status, where the comparator included both non-DM patients and DM non-metformin users, we extracted data based on a DM non-metformin comparator in preference. Where a time-varying covariate was used to model treatment effect, the most conservative HR was selected. Where reported, the HR after adjustment for potential confounding factors was extracted in preference to an unadjusted value. Since all eligible studies were of cohort design, the Newcastle–Ottawa quality assessment scale for cohort studies (NOS) [33] was used to evaluate methodological quality.

### statistical analysis

HRs and associated statistics were either extracted directly from the study reports or estimated from the Kaplan–Meier curves or summary statistics using published methods [34–36]. Where sufficient data were available on outcomes for individual cancer types, a meta-analysis was conducted with a primary outcome of RFS and secondary outcomes of OS and CSS. HRs were combined across trials using a fixed-effect model. Heterogeneity was assessed using the *χ*^2^ test and the *I*^2^ statistic. A random-effects model (DerSimonian and Laird) [37] was used to assess whether the results were robust to the choice of model. Probability values were two-sided, with *P* < 0.05 considered of statistical significance.

We also preplanned analyses to explore whether the size or the direction of the effect of metformin therapy varied according to specific study or patient characteristics, including: DM status of the comparator group (with and without non-DM patients in the comparator group), prostate cancer primary treatment type (prostatectomy or radical radiotherapy) and study design. The resulting HR estimates from study group analyses were compared using the *χ*^2^ test for interaction. We also planned to explore the impact of metformin dose/exposure on the outcomes described above where available. We also conducted unplanned sensitivity analyses for the primary outcome of RFS where at least two studies were available after restrictions. This was carried out according to study quality (restricted to studies with an NOS score ≥ the median); publication type (restricted to studies where a full publication was available); setting (restricted to hospital-based studies); follow-up (restriction of follow-up <3 years); and by the potential confounding factors accounted for (restricted to studies that adjusted for BMI, age, gender, cancer-specific prognostic factors and other DM medications). An additional unplanned exploratory analysis was also conducted according to whether the study was from a Western (North America or Europe) or non-Western population after a wide geographical distribution of studies was noted. Study group and sensitivity analyses were only conducted where study numbers were sufficient to be meaningful. Statistical analyses were carried out using STATA version 14.

## results

**Figure 1.**
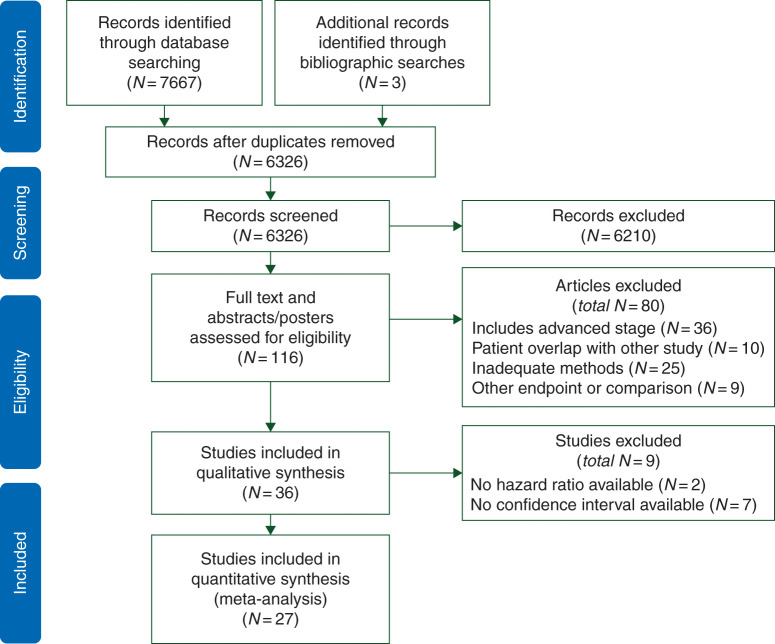
PRISMA study selection diagram.

After screening 7670 reports and conference abstracts, we identified 23 full publications and 4 conference abstracts that met our eligibility criteria, comprising 24 178 participants [38–64]. All were retrospective cohort studies except for one prospective cohort study embedded in a clinical trial [41]. The PRISMA study selection diagram is shown in Figure 1. The majority of identified studies examined the effect of metformin in one of four tumour types: prostate, colorectal, breast and urothelial cancer, which, therefore, represent the main focus of this analysis. A summary of the main characteristics for studies of breast, colorectal and prostate cancer is presented in Table 1, and a table of study characteristics for other cancer types is presented in Table 2.

**Table 1:** Main study characteristics: colorectal, prostate and breast cancer

Tumour group	Study author	Patient characteristics		Study characteristics				Comparator DM status		Outcomes			Definition of metformin exposure	Median follow-up (months)	Potential confounders (R = reported and not significant, M = included in multivariate model, x = not assessed, or significant but not adjusted for)					NOS score
		Treatment	Stage/other restrictions	Sample size *(met/total)*	Article type	Study location	Setting (H = Hospital, P = Population)	DM	Non-DM	RFS	OS	CSS			BMI	Age	Sex	Cancer- specific variables	Other DM meds	
Colorectal adenocarcinoma	Spillane [38]	Not specified	I–III	*207/315*	Full	Ireland	P	✓	X	X	✓	✓	In year before diagnosis	46	X	M	M	M	M	7
	Lee, GE [39]	Not specified	II–III	*223/356*	Abstract	Singapore	H	✓	X	✓	✓	X	At diagnosis	78	X	M	X	M	X	5
	Lee, JH [40]	Not specified	III^a^	*96/220*	Full	Korea	H	✓	X	X	✓	✓	>6 m exposure	41	M^b^	M^b^	M^b^	M^b^	M^b^	8
	Singh [41]	Not specified	III /colon only	*115/267*	Abstract	USA and Canada	H	✓	X	✓	✓	X	Before randomisation	Not given	X	M	M	M	X	5
	Zanders [42]	Not specified	I–III	*512/778*	Full	The Netherlands	P	✓	X	X	✓	X	Cumulative exposure	41	X	M	M	M	M	7
Prostate adenocarcinoma	Allott [43]	Prostatectomy	Localised	*155/369*	Full	USA	H	✓	X	✓	X	✓	At surgery	59/73^c^	M	M	n/a	M	X	8
	Kaushik [44]	Prostatectomy	Localised	*323/885*	Full	USA	H	✓	X	✓	✓	X	In 3 months before surgery	61	M	M	n/a	M	R	7
	Rieken WJU [45]	Prostatectomy	Localised	*287/6486*	Full	USA and Europe	H	X	✓	✓	X	X	At surgery	25	X	M	n/a	M	n/a	6
	Spratt [46]	Radical radiotherapy	Localised	*157/319*	Full	USA	H	✓	X	✓	✓	✓	At diagnosis or after radiotherapy	104	R	M	n/a	M	R	8
	Margel [47]	Prostatectomy or radical radiotherapy	Localised^a^/ ≥66 years old	*Total 955*	Full	Canada	P	✓	X	X	✓	✓	Cumulative exposure	56	X	M	n/a	M	M	8
	Zannella [48]	Radical radiotherapy	Localised	*114/504*	Full	Canada	H	✓	✓	✓	X	X	At the time of radiotherapy	82	X	R	n/a	M	X	5
	Danzig [49]	Prostatectomy	Localised	*98/767*	Full	USA	H	✓	X	✓	X	X	At surgery	27	X	M	n/a	M	X	6
	Taira [50]	Brachytherapy	Localised	*126/2298*	Full	USA	H	✓	✓	X	✓	X	Diagnosis to 3 months after brachytherapy	100	M	M	n/a	M	X	7
Breast adenocarcinoma	Oppong [51]	Adjuvant chemo	I–III	*76/141*	Full	USA	H	✓	X	✓	✓	X	Diagnosis to 6 months after	87	R	M	n/a	M	M	8
	Bayraktar [52]	Adjuvant chemo	I–III/triple negative	*63/130*	Full	USA	H	✓	X	✓	✓	X	During adjuvant chemo	62	M^^d^^	M	n/a	M	R	8
	Lega [53]	Breast cancer surgery	Infer I–III/≥66 years	*868/1774*	Full	Canada	P	✓	X	X	✓	✓	Cumulative exposure	54	X	M	n/a	M	M	6

NOS, Newcastle–Ottawa Quality Assessment Scale for Cohort Studies; BMI, body mass index; met, metformin; N/A, not applicable; RFS, recurrence-free survival; OS, overall survival; CSS, cancer-specific survival.

^a^Data from subanalysis.

^b^Main analysis only.

^c^Metformin/non-metformin.

^d^Adjustment for body weight.

**Table 2:** Main study characteristics: other cancer types

Tumour group	Study author	Patient characteristics		Study characteristics				Comparator DM status		Outcomes			Definition of metformin exposure	Median follow-up (months)	Potential confounders (R = reported & not significant, M = included in multivariate model X = not assessed, or significant but not adjusted for)					NOS Score
		Treatment	Stage/other restriction	Sample size (*met/total*)	Article type	Study location	Setting (H = hospital, P = population)	DM	Non-DM	RFS	OS	CSS			BMI	Age	Sex	Cancer specific	Other DM meds	
Urothelial carcinoma	Rieken BJU [54]	TURBT	pTa–pT1 N0 M0 /urothelial carcinoma of bladder (NMI)	*43*/*1035*	Full	USA and Europe	H	X	✓	✓	✓	X	At surgery	64	X	M	R	M	n/a	8
	Rieken UO [55]	Radical surgery	M0 /invasive urothelial carcinoma of bladder	*80*/*1382*	Full	USA and Europe	H	X	✓	✓	✓	✓	At diagnosis	34	M	M	M	M	n/a	8
	Rieken EJS [56]	Radical surgery	M0/upper tract urothelial carcinoma	*194*/*2330*	Full	USA, Europe and Japan	H	X	✓	✓	✓	✓	At surgery	36	X	M	M	M	n/a	6
Head and neck (squamous cell carcinoma)	Kwon [57]	Curative surgery or radiotherapy	No distant metastases	*99*/*1072*	Full	Korea	H	X	✓	✓	✓	✓	Ever exposure	65	M	M	R	M	n/a	8
	Thompson [58]	Not specified	Disease-free at 3 months/oral-oropharynx	*33*/*78*	Full	USA	H	✓	X	✓	X	X	Diagnosis to relapse	44	X	R	R	R	X	5
Renal cell carcinoma	Hakimi [59]	Partial/radical nephrectomy	T2–T3 N0 M0	*55*/*784*	Full	USA	H	✓	✓	✓	X	✓	At surgery	41	M	M	R	M	X	6
	Psutka [60]	Partial/radical nephrectomy	Localised	*83*/*200*	Full	USA	H	✓	X	✓	✓	✓	In 90 days before surgery	97	R	M	R	M	X	8
Pancreatic adenocarcinoma	Ambe [61]	Radical surgery	Resectable	*19*/*44*	Abstract	USA	H	✓	X	X	✓	X	At surgery	Not given	R	R	R	R	X	7
Non-small-cell lung carcinoma	Fortune-Greeley [62]	Not specified	data on stage I–II	*Not given*	Abstract	USA	H	✓	X	X	✓	X	Not given	Not given	M	M	X	M	X	6
Endometrial cancer	Ko [63]	Not specified	I–IV (RFS data extracted)	*200*/*363*	Full	USA	H	✓	X	✓	X	X	At diagnosis	33	R	M	n/a	M	R	8
Gastric cancer	Lee, CK [64]	Gastrectomy	I–III	*132*/*326*	Full	Korea	H	✓	X	✓	✓	✓	Cumulative exposure	74	M	M	M	M	M	9

NOS, Newcastle–Ottawa Quality Assessment Scale for Cohort Studies; BMI, body mass index; met, metformin; N/A, not applicable; NMI, non-muscle invasive; TURBT, transurethral resection of bladder tumour; RFS, recurrence-free survival; OS, overall survival; CSS, cancer-specific survival.

### colorectal cancer

**Figure 2.**
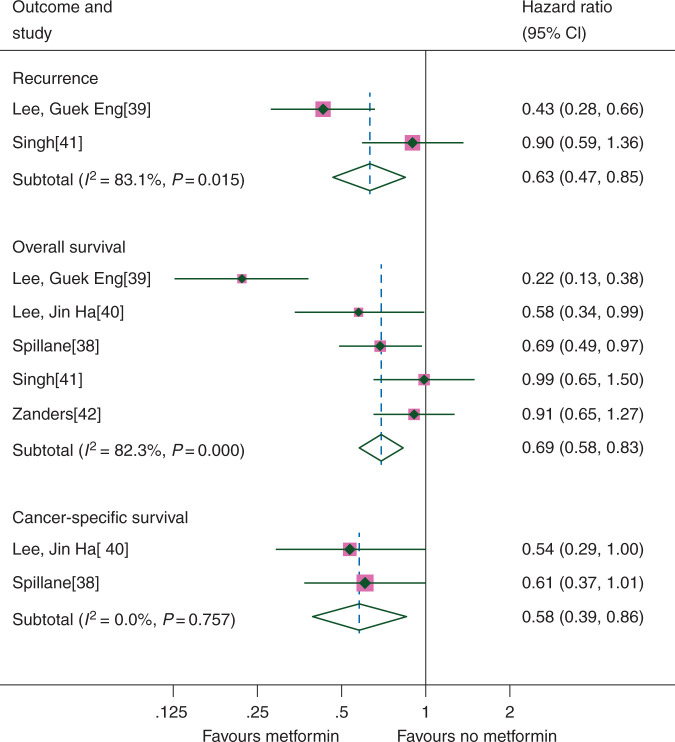
Colorectal cancer outcomes according to metformin use.

RFS was assessed in two studies (623 patients), OS in five studies (1936 patients) and CSS in two studies (535 patients). Overall, metformin use appeared to demonstrate significant improvements in RFS (HR 0.63, CI 0.47–0.85), OS (HR 0.69, CI 0.58–0.83) and CSS (HR 0.58, CI 0.39–0.86) (Figure 2), although there was variation between the results of the individual studies for RFS (*I*^2^ = 83.1%, *P* = 0.015) and OS (*I*^2^ = 82.3 *P* < 0.001). When the random-effects model was applied, the benefits seen for both OS (HR 0.62, CI 0.40–0.97) and CSS (HR 0.58, CI 0.39–0.86) remained, but there was no longer a significant benefit of metformin on RFS (HR 0.62, CI 0.30–1.29). In an unplanned exploratory analysis that grouped studies with Western and non-Western populations separately, we found there was a significant interaction between the effect of metformin on OS and the population studied (*χ*^2^ = 14.31, *P* < 0.001). In studies in non-Western populations, there was a highly significant benefit of metformin on OS (HR 0.36, CI 0.25–0.53); however, there was evidence of heterogeneity (*I*^2^ = 85.8%, *P* = 0.013). In studies with Western populations, only a trend towards a significant effect was identified (OS HR 0.84, CI 0.68–1.03) with no clear evidence of heterogeneity (*I*^2^ = 4.6%, *P* = 0.350). In unplanned sensitivity analyses, there appeared to be a larger relative benefit of metformin on OS when analyses were restricted to studies that had follow-up of >3 years (HR 0.64, CI 0.52–0.78). Further details of study group and sensitivity analyses for all tumour types are available in supplementary Table S1, available at *Annals of Oncology* online.

### prostate cancer

**Figure 3.**
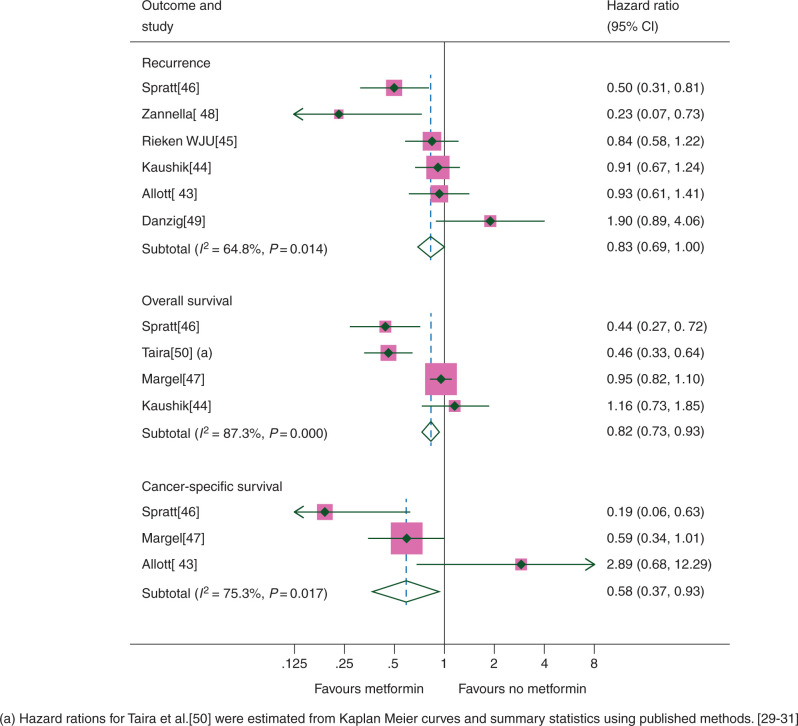
Prostate cancer outcomes according to metformin use.

RFS was assessed in six studies (9330 patients), OS in four studies (4457 patients) and CSS in three studies (1643 patients). Metformin use demonstrated a borderline significant improvement in RFS (HR 0.83, CI 0.69–1.00), and significant improvements in OS (HR 0.82, CI 0.73–0.93) and CSS (HR 0.58, CI 0.37–0.93) (Figure 3); however, the relationship was inconsistent across studies (RFS *I*^2^ = 64.8%, *P* = 0.014; OS *I*^2^ = 87.3%, *P* < 0.001; CSS *I*^2^ = 75.3%, *P* = 0.017), which was reflected when the random-effects model was applied (RFS HR 0.80, CI 0.57–1.13; OS 0.69, CI 0.44–1.10; CSS 0.64, CI 0.19–2.12).

**Figure 4.**
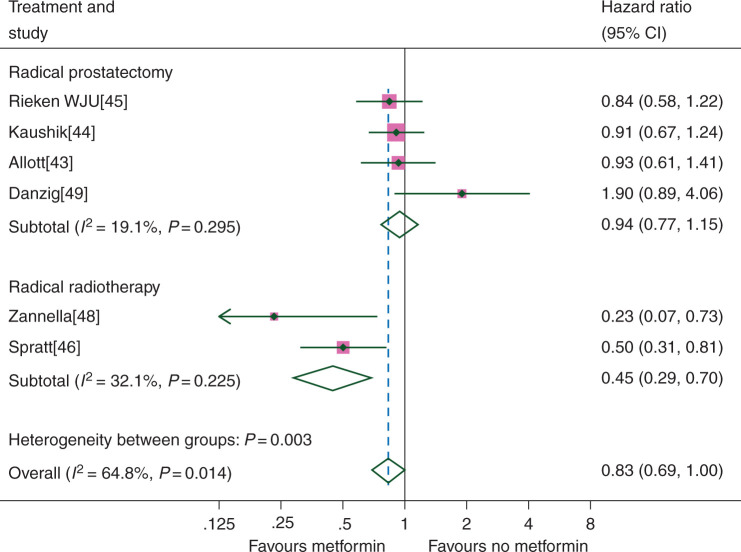
Prostate cancer recurrence-free survival according to metformin use for different treatment groups.

In a pre-specified analysis, there was significant interaction between the effect of metformin and the primary treatment type on RFS (*χ*^2^ test for interaction 9.03, *P* = 0.003). For patients receiving radical radiotherapy [46, 48], there was a significant benefit from metformin (HR 0.45, CI 0.29–0.70), whereas no significant benefit was seen for patients who underwent radical prostatectomy (HR 0.94, CI 0.77–1.15) (Figure 4). Only a single study was able to provide data on OS and CSS in those having radical radiotherapy; however, significant improvements were seen in both (OS 0.44, CI 0.27–0.72; CSS 0.19, CI 0.06–0.63) [46]. We found no evidence of an interaction between the effect of metformin on RFS and the presence or absence of non-DM patients in the comparator group (*χ*^2^ 0.49, *P* = 0.48).

In unplanned sensitivity analyses, there appeared to be a larger relative benefit of metformin on RFS when analyses were restricted to studies that had a follow-up of >3 years (HR 0.77, CI 0.62–0.96) or considered other DM medications in their analysis (HR 0.79, CI 0.64–0.98).

### breast cancer

**Figure 5.**
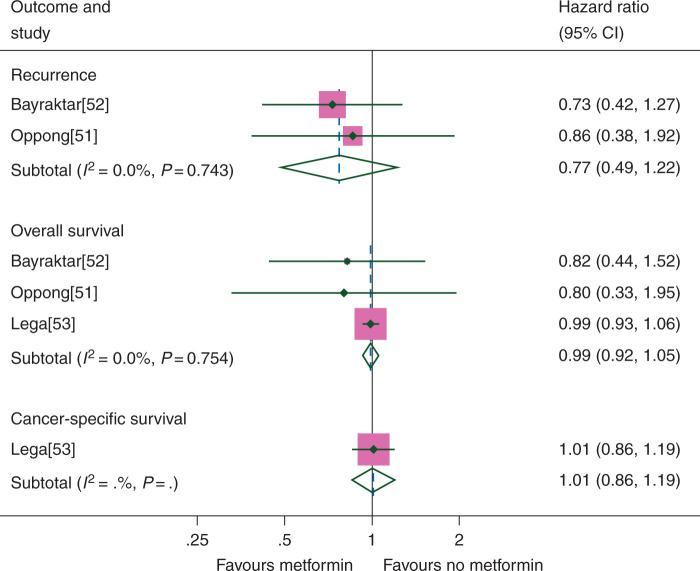
Breast cancer outcomes according to metformin use.

RFS was assessed in 2 studies containing 271 patients and OS in 3 studies including 2045 patients. Metformin demonstrated a trend towards improvement in RFS (HR 0.77, CI 0.49–1.22) (Figure 5); however, no effect was seen in OS (HR 0.99, CI 0.92–1.05). There was no evidence of variation between the results of the studies either for RFS (*I*^2^ = 0.0%, *P* = 0.74) or OS (*I*^2^ = 0.0%, *P* = *0*.*75*). As CSS was only available for one study containing 1774 patients, no meta-analysis was possible for this outcome; however in this study, metformin did not appear to have an impact on CSS (HR 1.01, CI 0.86–1.19). There were insufficient study numbers for any meaningful study group or sensitivity analyses.

### urothelial cancer

**Figure 6.**
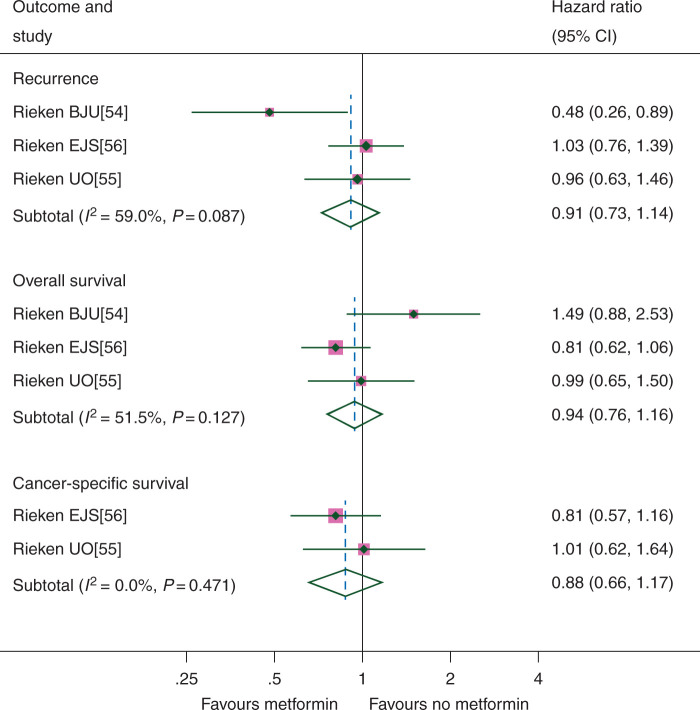
Urothelial cancer outcomes according to metformin use.

Studies included patients with upper tract urothelial carcinoma and urothelial carcinoma of the bladder. RFS was assessed in 3 studies including 4747 patients, and OS in 3 studies including 4747 patients, of which 2 also assessed CSS including 3712 patients. There was no clear evidence that metformin improved either RFS (HR 0.91, CI 0.73–1.14), OS (HR 0.94, CI 0.76–1.16) or CSS (HR 0.88, CI 0.66–1.17) (Figure 6). Although there was some evidence of inconsistency between the results of studies for both RFS (*I*^2^ = 59.0%, *P* = 0.087) and OS (*I*^2^–51.5%, *P* = 0.127), the results did not change significantly when the random-effects model was applied (RFS HR 0.84, CI 0.57–1.24; OS HR 1.00, CI 0.72–1.39; CSS HR 0.88, CI 0.66–1.17). There were insufficient study numbers for any meaningful study group or sensitivity analyses.

### other cancer types

There were insufficient studies identified to warrant meta-analyses for other cancer types, the findings of which are presented in Table 3. In head and neck cancer, a positive trend towards improved RFS and CSS was seen in one study [57], but there was no effect on OS. However, the second study identified showed a potential detriment of metformin use on RFS [58]. In renal cell carcinoma, two studies were identified, both showing a non-significant inverse relationship with metformin use and RFS, and no significant benefit in OS or CSS. Single studies were identified showing a significant improvement in OS in lung cancer, RFS and OS in endometrial cancer and RFS, OS and CSS in gastric cancer. A small single study in pancreatic cancer did not suggest any effect of metformin; however, this study had a very small sample size.

**Table 3:** Cancer outcomes by metformin use for tumour types with limited numbers of studies

Tumour group	Study author	Sample size	Recurrence-free survival HR (95% CI)	Overall survival HR (95% CI)	Cancer-specific survival HR (95% CI)
Head and neck	Kwon [57]	1072	0.76 (0.49–1.21)	0.95 (0.59–1.50)	0.79 (0.42–1.50)
	Thompson [58]	78	1.26 (0.62–2.56)	—	—
Renal cell carcinoma	Hakimi [59]	784	1.22 (0.66–2.27)	—	0.76 (0.21–2.70)
	Psutka [60]	200	1.07 (0.61–1.88)	0.74 (0.48–1.15)	0.83 (0.41–1.67)
Pancreas	Ambe [61]	44	—	0.54 (0.16–1.68)	—
Lung	Fortune [62]	Not given by stage	—	0.85 (0.77–0.93)	—
Endometrial	Ko [63]	363	0.56 (0.34–0.91)	0.43 (0.24–0..77)	—
Gastric	Lee, CK [64]^a^	326	0.86 (0.80–0.94)	0.87 (0.80–0.95)	0.87 (0.78–0.96)

^a^HR for each 6 months of metformin use.

### duration and dose

The impact of different exposures to metformin on early-stage cancer outcomes is examined in some of the identified studies; however, limited data and differences in the methods used to investigate exposure preclude any study-group analyses. In colorectal cancer, Spillane et al. [38] conducted additional analyses on dose intensity and found survival benefits for high-intensity metformin users not using other diabetic therapies (CSS HR 0.44, CI 0.20–0.95; OS HR 0.41, CI 0.24–0.70), but no significant benefits were identified in other subgroups. In gastric cancer, Lee et al. [64] found that increased cumulative duration of metformin use improved cancer-specific and all-cause mortality. Single studies in colorectal [42] and prostate cancer [43] also investigated the impact of different exposures to metformin but found no significant associations.

## discussion

Our analysis suggests that metformin could be a useful adjuvant agent, particularly in colorectal and prostate cancer. The number of studies identified for each tumour type is likely to reflect the incidence and demographics of the disease, particularly the likelihood of presentation with early-stage disease and a diagnosis of DM.

The variation in the adjuvant effects of metformin according to tumour type could be explained by differences in both patient characteristics and tumour biology. The effect of metformin on AMPK signalling has been hypothesised to be a major pathway through which metformin exerts its anti-cancer effects [10]. AMPK signalling dysregulation is also associated with metabolic syndrome [65], a cluster of conditions which include raised fasting glucose, dyslipidaemia, high blood pressure and central obesity [66]. Metabolic syndrome is also known to increase the risk of developing some cancers, particularly colorectal cancer [67], where it is also associated with poorer recurrence and survival outcomes [68]. In addition, it is known to develop as a consequence of androgen deprivation therapy in men with prostate cancer [69]. Metformin may improve OS by reducing the number of cardiovascular deaths associated with metabolic syndrome; however, the improvements in RFS and CSS identified suggest a direct anti-cancer effect. In prostate cancer, our study group analysis suggests that the beneficial effects of metformin use could be limited to those undergoing radical radiotherapy. The AMPK pathway is known to play a role in regulating cellular responses to radiotherapy, [70] and studies in xenograft mice models suggest that metformin can improve tumour oxygenation and therefore radiation response [71].

The limitations of our meta-analysis include the inherent weaknesses of observational data, particularly potential measurement errors in the exposure to metformin, and variation in the definition of metformin use, and the risk of time-related biases [72]. A high degree of variation between the results of studies was observed for a number of the outcomes investigated in most of the cancer types. Our sensitivity analyses were designed to explore possible reasons for this to inform future observational and clinical trial design; however, only a small number of analyses were possible due to insufficient study numbers. For both prostate and colorectal cancer, the relative effect size appeared to increase for studies with follow-up of 3 years or greater, highlighting the importance of ensuring adequate duration of follow-up in future studies. Similarities have been seen in studies of aspirin, where greater benefits have been seen with longer follow-up [73–75]. A limited number of studies investigated the relation with frequency, dose and duration of metformin in early-stage cancer; however, findings are inconsistent and further research is required to better understand this relationship.

Previous studies have suggested that a diagnosis of DM has a negative impact on cancer outcomes [76, 77]; therefore, inclusion of non-DM patients in comparator groups could underestimate the beneficial effect of metformin. Owing to insufficient study numbers, it was only possible to analyse the effect of the presence or absence of non-DM patients in the comparator group for RFS in prostate cancer, where no evidence for an effect was found.

Other meta-analyses have investigated the effect of metformin on survival outcomes, across all stages of cancer, in individual tumour types, the findings of which are presented in supplemen-tary Table S2, available at *Annals of Oncology* online. In colorectal cancer, four meta-analyses have examined the effect on OS [21–24], two of which also investigated colorectal CSS [23, 24]. All meta-analyses identified significant improvements in these end points, which is consistent with the findings of this study. For prostate cancer, findings are less consistent. Five meta-analyses have examined the effect of metformin on OS [22, 23, 25–27], two of which also investigated prostate CSS [25, 26]. Only two meta-analyses identified a significant benefit in OS [23, 25], with no benefit identified in prostate CSS. This differs from the findings of this study where significant benefits in OS and prostate CSS were identified, which could suggest that metformin is better suited to the adjuvant setting for prostate cancer. In breast cancer, four meta-analyses examined OS [21–23, 28], two of which investigated breast CSS. Two meta-analyses identified a significant benefit in OS [21, 23, 28], the other approached significance (HR 0.81, CI 0..64–1.04) [22] and the two meta-analyses investigating breast CSS also showed significant improvements [23, 28]. This differs from the findings of this study where no significant benefit in OS and breast CSS was identified. This could suggest that metformin may be effective in those with established breast cancer, which is consistent with the findings of breast cancer window studies where direct anti-tumour effects have been identified [13, 14].

Investigation of metformin in the primary prevention setting presents a number of challenges, where the balance between adverse effects and benefits is likely to be less favourable and difficult to detect in a clinical trial because of the low event rate. While the advanced setting can provide a sufficient event rate, there is evidence to suggest that metformin requires long-term use to exert its anti-cancer effect [78], and therefore, patients with established cancer with more limited prognoses may not be able to receive metformin long enough for a therapeutic benefit to emerge. Therefore, the adjuvant setting could be most suitable for investigating the anti-cancer effects of metformin.

### current trial activity

In colorectal cancer, a phase III trial of metformin versus standard care assessing recurrence and survival in stage III disease is now in set-up phase in South Korea (NCT02614339). In prostate cancer, the Metformin Active Surveillance Trial (NCT01864096), an ongoing randomised phase III trial of metformin versus placebo given before primary therapy is assessing time to progression in men with low-risk prostate cancer. The STAMPEDE trial (NCT00268476), a multi-arm multi-stage randomised, controlled trial investigating a number of agents in the treatment of hormone-naïve, high-risk, localised and metastatic prostate cancer, aims to evaluate whether the addition of metformin improves survival in this group. Recruitment to this comparison is due to open in autumn 2016. In breast cancer, our results did not identify any meaningful benefit of metformin use in the adjuvant setting; however, this could be due to the limited number of studies identified. Additional supporting data are available in the primary prevention and treatment setting (across all stages), where meta-analyses have shown a beneficial effect [21, 23, 28, 79]. A randomised phase III trial of metformin versus placebo assessing recurrence and survival in early-stage breast cancer has recently completed recruitment (MA-32, NCT01101438) and the results are awaited.

### conclusions

The findings of this meta-analysis support the concept of randomised clinical trials using metformin in the adjuvant setting, with the strongest supporting evidence in colorectal and prostate cancer, particularly those treated with radical radiotherapy. Such trials could also further our understanding of the relationships between cancer outcomes and the dose and duration of metformin. The authors are not aware of any ongoing adjuvant phase III trials of metformin in prostate cancer, or colorectal cancer in Western populations. In other tumour types, where there is currently less evidence, further observational studies are needed to advise suitability for investigation in any future randomised, controlled trials.

## Supplementary Material

Supplementary Data

Supplementary Data
