# Supplementary material for: Metformin as an adjuvant treatment for cancer: a systematic review and meta-analysis
Source: Ann Oncol. 2016 Sep 28;27(12):2184–95. doi: 10.1093/annonc/mdw410 (PMC5178140; doi:10.1093/annonc/mdw410)
Supplement: Supplementary Data [file mdw410_supplementary_data.zip › mdw410supp_table2.docx]

Supplementary table S2 – Results from other meta-analyses of the effect of metformin on survival outcomes across all stages for individual tumour types

| Tumour type **(endpoint)** | Meta-analysis  Effect size (95% confidence interval) | | | | | | | | | | | | |
| --- | --- | --- | --- | --- | --- | --- | --- | --- | --- | --- | --- | --- | --- |
|  | Zhang ZJ  (relative risk) | Lega | | Yin  (fixed effect) | | Mei | | Raval | Stopsack | | Yu | | Xu |
| Breast  **(OS)** | RR 0·70, (0·55-0·88) | HR 0·81, (0·64-1·04) | | HR 0·94,  (0·90-0·99) | | x | | x | x | | x | | HR 0·53, (0·39-0·71) |
| Colorectal  **(OS)** | RR 0·70,  (0·59-0·84) | HR 0·65, (0·56-0·76) | | HR 0·65, (0·56-0·77) | | HR 0·56,  (0·41-0·77) | | x | x | | x | | x |
| Prostate  **(OS)** | x | HR 0·73, (0·51-1·06) | | HR 0·68,  (0·51-0·90) | | x | | HR 0·86,  (0·67-1·10) | HR 0·88,  (0·86-0·90) | | HR 0·86,  (0·64-1·14) | | x |
|  | | | | | | | | | | | | | |
| Breast  **(CSS)** | x | x | HR 0·88,  (0·79-0·99) | | x | | x | | | x | | x | HR 0·89, (0·79-1·00) |
| Colorectal  **(CSS)** | x | x | HR 0·66, (0·50-0·87) | | HR 0·66,  (0·50-0·87) | | x | | | x | | x | x |
| Prostate  **(CSS)** | x | x | x | | x | | HR 0·76,  (0·43-1·33) | | | HR 0·76,  (0·44-1·31) | | x | x |

Statistically significant benefit No statisticially signficiant benefit found

OS= Overall survival, CSS= Cancer specific survival
