# Supplementary material for: Metformin as an adjuvant treatment for cancer: a systematic review and meta-analysis
Source: Ann Oncol. 2016 Sep 28;27(12):2184–95. doi: 10.1093/annonc/mdw410 (PMC5178140; doi:10.1093/annonc/mdw410)
Supplement: Supplementary Data [file mdw410_supplementary_data.zip › mdw410supp_data1.docx]

**Supplementary data S1 – Search Strategies**

**MEDLINE Search strategy**

**Interventions:**

1. exp Biguanides/
2. exp Metformin/
3. Metformin.tw,ot.
4. Biguanide$.tw,ot.
5. or/1-4 (Combines all of the intervention hits)

Outcomes (+ disease setting)

1. exp Mortality/
2. mortality.tw,ot.
3. mortaliti$.tw,ot.
4. or/6-8 (combines all mortality studies)
5. exp Primary Prevention/
6. exp Secondary Prevention/
7. (prevention$ or prevent$).tw,ot.
8. exp Neoplasm/
9. (cancer$ or neoplasm$ or tumo?r$).tw,ot.
10. or/10-14 (combines all primary and secondary prevention studies)

Standard RCT search

1. exp Randomized Controlled Trials as topic/
2. Randomized Controlled Trial.pt.
3. exp Controlled Clinical Trials as topic/
4. Controlled Clinical Trial.pt.
5. exp Random Allocation/
6. exp Double-Blind Method/
7. exp Single-Blind Method/
8. or/16-22

Other Study types

1. Epidemiologic Studies/
2. exp Case-Control Studies/
3. exp Cohort Studies/
4. Cross-Sectional Studies/
5. (epidemiologic adj (study or studies)).ab,ti.
6. case control.ab,ti.
7. (cohort adj (study or studies)).ab,ti.
8. cross sectional.ab,ti.
9. cohort analy$.ab,ti.
10. (follow up adj (study or studies)).ab,ti.
11. longitudinal.ab,ti.
12. retrospective$.ab,ti.
13. prospective$.ab,ti.
14. (observ$ adj3 (study or studies)).ab,ti.
15. adverse effect?.ab,ti.
16. Or/24-38 (Non-RCTs)
17. exp "Review Literature as topic"/
18. exp Technology Assessment, Biomedical/
19. exp Meta-analysis as topic/
20. Meta-analysis.pt.
21. hta.tw,ot.
22. (health technology adj6 assessment$).tw,ot.
23. (meta analy$ or metaanaly$ or meta?analy$).tw,ot.
24. ((review$ or search$) adj10 (literature$ or medical database$ or medline or pubmed or embase or cochrane or cinahl or psycinfo or psyclit or healthstar or biosis or current content$ or systemat$)).tw,ot.
25. or/40-47 (Combines all reviews)
26. 23 or 39 (RCTs or non-RCTs)
27. 48 or 49 (RCTs or non-RCTs or reviews)
28. 5 and 15 and 50 (Interventions + prevention studies + RCTs and non-RCTs and reviews)
29. 5 and 9 and 50 (Interventions and mortality + RCTs and non-RCTs and reviews)
30. 51 or 52 (Combines prevention and mortality)
31. limit 53 to animals
32. limit 53 to humans
33. 54 not 55 (Animals not humans)
34. 53 not 56 (Prevention and mortality – all studies excluding animal studies)

**EMBASE Search strategy**

Interventions:

1. exp Biguanides/
2. exp Metformin/
3. Metformin.tw,ot.
4. Biguanide$.tw,ot.
5. or/1-4 (Combines all of the intervention hits)

Outcomes (+ disease setting)

1. exp Mortality/
2. mortality.tw,ot.
3. mortaliti$.tw,ot.
4. or/6-8 (combines all mortality studies)
5. exp Primary Prevention/
6. exp Secondary Prevention/
7. (prevention$ or prevent$).tw,ot.
8. exp Neoplasm/
9. (cancer$ or neoplasm$ or tumo?r$).tw,ot.
10. or/10-14 (combines all primary and secondary prevention studies)

Standard RCT search

1. randomi$.tw
2. placebo.mp
3. double-blind.tw
4. or/16-18

Other Study types

1. epidemiology/
2. exp case control study/
3. exp cohort analysis/
4. cross sectional study/
5. (epidemiologic adj (study or studies)).ab,ti.
6. case control.ab,ti.
7. (cohort adj (study or studies)).ab,ti.
8. cross sectional.ab,ti.
9. cohort analy$.ab,ti.
10. (follow up adj (study or studies)).ab,ti.
11. longitudinal.ab,ti.
12. retrospective$.ab,ti.
13. prospective$.ab,ti.
14. (observ$ adj3 (study or studies)).ab,ti.
15. adverse effect?.ab,ti.
16. Or/20-34 (Non-RCTs)
17. exp literature/
18. exp biomedical technology assessment/
19. exp meta-analysis/
20. meta-analysis.kw
21. hta.tw,ot.
22. (health technology adj6 assessment$).tw,ot.
23. (meta analy$ or metaanaly$ or meta?analy$).tw,ot.
24. ((review$ or search$) adj10 (literature$ or medical database$ or medline or pubmed or embase or cochrane or cinahl or psycinfo or psyclit or healthstar or biosis or current content$ or systemat$)).tw,ot.
25. or/36-43 (Combines all reviews)
26. 19 or 35 (RCTs or non-RCTs)
27. 44 or 45 (RCTs or non-RCTs or reviews)
28. 5 and 15 and 46 (Interventions + prevention studies + RCTs and non-RCTs and reviews)
29. 5 and 9 and 46 (Interventions and mortality + RCTs and non-RCTs and reviews)
30. 47 or 48 (Combines prevention and mortality)
31. limit 49 to animals
32. limit 49 to humans
33. 50 not 51 (Animals not humans)
34. 49 not 52 (Prevention and mortality – all studies excluding animal studies)

**Cochrane search strategy**

#1 “metformin”

#2 “neoplasm”

#1 and#2

**ASCO and ESMO search strategy**

Keyword search for “metformin”

**Clinicaltrials.gov, ISRCTN and EU Clinical Trials Register search strategy**

Search term “metformin” AND “cancer”

Restricted to trials with published results
