# Supplementary material for: Metformin as an adjuvant treatment for cancer: a systematic review and meta-analysis
Source: Ann Oncol. 2016 Sep 28;27(12):2184–95. doi: 10.1093/annonc/mdw410 (PMC5178140; doi:10.1093/annonc/mdw410)
Supplement: Supplementary Data [file mdw410_supplementary_data.zip › mdw410supp_table1.docx]

**Supplementary table S1 – Sub group and sensitivity analyses**

| **Restriction** | **Study group** | **RFS** | **OS** | **CSS** |
| --- | --- | --- | --- | --- |
| **Colorectal cancer** | | | | |
| **All** | **HR** | Lee GE, Singh  HR=0·63 (0·47, 0·85), *p=0·002* | Lee GE, Lee GH, Spillane, Singh, Zanders  HR=0·69 (0·58, 0·83), *p<0·001* | Lee JH, Spillane  HR=0·58 (0·39, 0·86), *p=0·006* |
| **NOS<7 (median)** | **HR** | x | Lee GH, Spillane, Zanders  HR=0·75 (0·61, 0·94), *p=0·011* (hetero *p=0·301*) | x |
| **Without abstracts** | **HR** | x | Lee GH, Spillane, Zanders  HR=0·75 (0·61, 0·94), *p=0·011* (hetero *p=0·301*) | x |
| **Without western patients**  **(no North America, Europe)** | **Western population** | x | Western (Spillane, Singh, Zanders):  HR=0·839 (0·682, 1·033), *p=0·097* (hetero *p=0·350*) | x |
|  | **Non-western population** | x | Non-West (Lee GE, Lee GH):  HR=0·362 (0·247, 0·531), *p<0·001* (hetero *p=0·013*) | x |
|  | **Interaction** | x | Chi-squared=14·31, *p<0·001* | x |
| **Without population setting** | **HR** | x | Lee GE, Lee GH, Singh  Hr=0·58 (0·43, 0·76), *p<0·001* (hetero *p<0·001* | x |
| **<36 months** | **HR** | x | Lee GE, Lee GH, Spillane, Zanders  HR=0·64 (0·52, 0·78), *p<0·001* (hetero *p<0·001*) | x |
| **Sex** | **HR** | x | Lee GH, Spillane, Singh, Zanders  HR=0·80 (0·66, 0·97), *p=0·023* (hetero *p=0·295*) | x |
| **Other DM Meds** | **HR** | x | Lee GH, Spillane, Zanders  HR=0·75 (0·61, 0·94), *p=0·011* (hetero *p=0·301*) | x |
| **Prostate cancer** | | | | |
| **All** | **HR** | Spratt, Zanella, Rieken WJU, Kaushik, Allott, Danzig  HR=0·83 (0·69, 1·00), *p=0·044* | Spratt, Taira, Margel, Kaushik  HR=0·82 (0·73, 0·94), *p=0·003* | Spratt, Margel, Allott  HR=0·58 (0·37, 0·93), *p=0·023* |
| **Comparator group** | **DM only control** | Spratt, Kaushik, Allott, Danzig  HR=0·863 (0·698, 1·066), *p=0·171* (hetero *p=0·025*) | x | x |
|  | **Mixed or non-DM control** | (Zannella, Rieken WJU)  HR=0·744 (0·522, 1·060), *p=0·102* (hetero *p=0·037*) | x | x |
|  | **Interaction** | Chi-squared = 0·49, p=0·483 | x | x |
| **NOS<7 (median)** | **HR** | Spratt, Kaushik, Allott  HR=0·807 (0·647, 1·006), *p=0,057* (hetero *p=0·089*) | x | x |
| **Without population setting** | **HR** | x | Spratt, Taira, Kaushik  HR=0·577 (0·455, 0·731), *p<0·001* (hetero *p=0·003*) | Spratt, Allott  HR=0·570 (0·227, 1·431), *p=0·231* (hetero *p=0·004*) |
| **<36 months** | **HR** | Spratt, Zanella, Kaushik, Allott  HR=0·772 (0·622, 0·959), *p=0·019* (hetero *p=0·027*) | x | x |
| **BMI** | **HR** | Spratt, Kaushik, Allott,  HR=0·807 (0·647, 1·006), *p=0,057* (hetero *p=0·089*) | Spratt, Taira, Kaushik  HR=0·577 (0·455, 0·731), *p<0·001* (hetero *p=0·003*) | Spratt, Allott  HR=0·570 (0·227, 1·431), p=0·231 (hetero *p=0·004*) |
| **Other DM Meds** | **HR** | Spratt, Rieken WJU, Kaushik  HR=0·788 (0·637, 0·975), *p=0·028* (hetero *p=0·111*) | Spratt, Margel, Kaushik  HR=0·910 (0·793, 1·044), *p=0·178* (hetero *p=0·008*) | Spratt, Margel  HR=0·487 (0·299, 0·795) (hetero *p=0·089*) |

| **Restriction** | **Study group** | **RFS** | **OS** | **CSS** |
| --- | --- | --- | --- | --- |
| **Breast cancer** | | | | |
| **All** | **HR** | Bayraktar, Oppong  HR=0·77 (0·49, 1·22), *p=0·263* | Bayraktar, Oppong, Lega  HR=0·99 (0·93, 1·05) | Lega  HR=1·01 (0·86, 1·19), *p=0·907* |
| **NOS<7 (median)** | **HR** | x | Bayraktar, Oppong  HR=0·81 (0·49, 1·35), *p=0·426* (hetero *p=0·964* | x |
| **Without population setting** | **HR** | x | Bayraktar, Oppong  HR=0·81 (0·49, 1·35), *p=0·426* (hetero *p=0·964* | x |
| **BMI** | **HR** | x | Bayraktar, Oppong  HR=0·81 (0·49, 1·35), *p=0·426* (hetero *p=0·964* | x |
| **Urothelial cancer** | | | | |
| **All** | **HR** | Reiken BJU, Reiken EJS, Reiken UO  HR=0·91 (0·73, 1·14), *p=0·414* | Reiken BJU, Reiken EJS, Reiken UO  HR=0·94 (0·76, 1·16), *p=0·549* | Reiken EJS, Reiken UO  HR=0·88 (0·66, 1·17), *p=0·361* |
| **NOS<7 (median)** | **HR** | Reiken BJU, Reiken UO  HR=0·77 (0·54, 1·09), *p=0·140* (hetero *p=0·068*) | Reiken BJU, Reiken UO  HR=1·16 (0·84, 1·61), *p=0·378* (hetero *p=0·234*) | x |
| **<36 months** | **HR** | Reiken BJU, Reiken EJS  HR=0·89 (0·68, 1·17), *p=0·397* (hetero *p=0·029*) | Reiken BJU, Reiken EJS  HR=0·92 (0·72, 1·17), *p=0·506* (hetero *p=0·044*) | x |

Footnote: Study group analyses are only presented where there are two or more studies in each group.

Sensitivity analyses are only presented where there are two or more studies after restriction.

The fixed effect model is used for all analyses.
