# Supplementary material for: Metformin as an adjuvant treatment for cancer: a systematic review and meta-analysis
Source: Ann Oncol. 2016 Sep 28;27(12):2184–95. doi: 10.1093/annonc/mdw410 (PMC5178140; doi:10.1093/annonc/mdw410)
Supplement: Supplementary Data [file mdw410_supplementary_data.zip › mdw410supp_data2.docx]

**Supplementary data S2 – Data extraction fields**

**Study characteristics**

- Tumour group
- Tumour subtype/histopathology
- Treatment
- Other eligibility restrictions
- Stage
- Comparator diabetic status
- Study design
- Cancer outcomes reported
- Publication type
- Study time period
- Countries
- Clinical setting (population level, hospital, number of centres)
- Metformin exposure definition
- Data source

**Participant characteristics**

- Mean/median age of participants
- Median follow-up
- Gender (%male)
- Median BMI
- Proportion of metformin users (%)
- Number of metformin users (intervention group)
- Number of non-metformin users (comparator group)

**Cancer outcome data**

- End-point terminology used
- Hazard ratio
- 95% confidence interval
- p-value

**Study confounding factors/biases**

- Covariates adjusted for
- Assessment of specific potential confounders
  - (BMI, age, gender, Diabetic status, tumour specific prognostic variables)
- List of potential biases
