# Supplementary material for: Metformin as an adjuvant treatment for cancer: a systematic review and meta-analysis
Source: Ann Oncol. 2016 Sep 28;27(12):2184–95. doi: 10.1093/annonc/mdw410 (PMC5178140; doi:10.1093/annonc/mdw410)
Supplement: Supplementary Data [file supp_27_12_2184__index.html]

Metformin as an adjuvant treatment for cancer: a systematic review and meta-analysis — Supplementary Data 

# Metformin as an adjuvant treatment for cancer: a systematic review and meta-analysis

## Supplementary Data

Supplementary Data

- Supplementary Data 1 - docx file
- Supplementary Data 2 - docx file
- Supplementary Table 1 - docx file
- Supplementary Table 2 - docx file
